# Explaining Diversity in Metagenomic Datasets by Phylogenetic-Based Feature Weighting

**DOI:** 10.1371/journal.pcbi.1004186

**Published:** 2015-03-27

**Authors:** Davide Albanese, Carlotta De Filippo, Duccio Cavalieri, Claudio Donati

**Affiliations:** 1 Department of Computational Biology, Research and Innovation Centre, Fondazione Edmund Mach, San Michele all'Adige, Italy; 2 Department of Food Quality and Nutrition, Research and Innovation Centre, Fondazione Edmund Mach, San Michele all'Adige, Italy; United States of America

## Abstract

Metagenomics is revolutionizing our understanding of microbial communities, showing that their structure and composition have profound effects on the ecosystem and in a variety of health and disease conditions. Despite the flourishing of new analysis methods, current approaches based on statistical comparisons between high-level taxonomic classes often fail to identify the microbial taxa that are differentially distributed between sets of samples, since in many cases the taxonomic schema do not allow an adequate description of the structure of the microbiota. This constitutes a severe limitation to the use of metagenomic data in therapeutic and diagnostic applications. To provide a more robust statistical framework, we introduce a class of feature-weighting algorithms that discriminate the taxa responsible for the classification of metagenomic samples. The method unambiguously groups the relevant taxa into clades without relying on pre-defined taxonomic categories, thus including in the analysis also those sequences for which a taxonomic classification is difficult. The phylogenetic clades are weighted and ranked according to their abundance measuring their contribution to the differentiation of the classes of samples, and a criterion is provided to define a reduced set of most relevant clades. Applying the method to public datasets, we show that the data-driven definition of relevant phylogenetic clades accomplished by our ranking strategy identifies features in the samples that are lost if phylogenetic relationships are not considered, improving our ability to mine metagenomic datasets. Comparison with supervised classification methods currently used in metagenomic data analysis highlights the advantages of using phylogenetic information.

## Author Summary

In metagenomics, the composition of complex microbial communities is characterized using Next Generation Sequencing technologies. Thanks to the decreasing cost of sequencing, large amounts of data have been generated for environmental samples and for a variety of health-associated conditions. In parallel there has been a flourishing of statistical methods to analyze metagenomic datasets, concentrating mainly on the problem of assessing the existence of significant differences between microbial communities in different conditions. However, for a large number of therapeutic and diagnostic applications it would be essential to identify and rank the microbial taxa that are most relevant in these comparisons. Here we present PhyloRelief, a novel feature-ranking algorithm that fills this gap by integrating the phylogenetic relationships amongst the taxa into a statistical feature weighting procedure. Without relying on a precompiled taxonomy, PhyloRelief determines the lineages most relevant to the diversification of the samples guided by the data. As such, PhyloRelief can be applied both to cases in which sequences can be classified according to a known taxonomy, and to cases in which this is not feasible, a common occurrence in metagenomic data analysis given the increasing number of new and uncultivable taxa that are discovered using these technologies.

## Introduction

Thanks to the possibility to characterize microbial communities through next generation sequencing, microbial ecology has become a central topic in many environmental and therapeutic applications. Extensive explorative studies of the microbiota colonizing several districts of the human body have been conducted, highlighting the large variability from site to site, as well as the interpersonal differences in the same body site [1]. The more extensively studied district is the human gastrointestinal tract (GI), whose metagenomics composition appears to be influenced by several factors [2], including age [3,4], geography [5], diet [6], and lifestyle [7]. In addition, a correlation between imbalances or abnormal composition of the gut microbiota and a number of pathologic conditions has been proposed. These alterations might be due to therapeutic interventions, like antibiotic treatment [8], or different lifestyle [9].

The growing body of evidence of the importance of the gut microbiota for the self-sustainability of health of the “holobiont” is opening the debate on the design of therapeutic intervention strategies. Fecal transplantation has shown its effectiveness and safety in the treatment of recurrent *Clostridium difficile* infections [10], which are known to correlate with altered microbiomes following antibiotic treatment [11]. Alternatives for bioremediation of microbiota alterations is the supplementation of pro- or prebiotics, while it has been suggested that antibiotic treatment and vaccination can be used to guide the structure of the gut microbiota towards a status that is compatible with health [12,13]. Most of these intervention strategies would greatly increase their efficacy using a precise definition of the microbial species that are differentially distributed in health and disease conditions. This task faces several difficulties. On one hand, most of the microorganisms composing the human and environmental microbiota are poorly characterized, difficult to cultivate, and lack a precise taxonomic classification. On the other hand, methods to unambiguously define the microbial taxa that are responsible for these differences are still lacking, and their identification usually relies on a small number of arbitrarily chosen association tests with high-level taxonomic classes, or on statistical learning methods, both evaluating only taxa for which a taxonomic classification is possible [14]. In addition, the low abundance of most microbial taxa in metagenomic samples poses additional challenges only recently tackled with statistical methods [15].

In amplicon metagenomics, the composition in term of microbial genera of a sample is inferred from the high throughput sequencing of a small number of diagnostic genomic loci, the most popular being the V1–V6 variable regions of the 16S rDNA gene for bacteria [16] and the ITS spacer for fungi [17], selectively amplified using broadly conserved PCR primers. As a proxy for species, Operational Taxonomic Units (OTUs) are determined by the clustering of the sequences up to a given level of similarity, usually 97%. Using the OTUs abundances, the differentiation between samples or classes of samples is accomplished by measuring their *β*-diversity, *i*.*e*. the variations in community membership across the different groups [18]. Given that the sequences of marker genes are available, phylogenetic measures of diversity such as UniFrac [19,20] have proven to be able to identify subtle differences in the structures of microbial communities by weighting species abundances with the phylogenetic relationships amongst taxa.

Here we present PhyloRelief, a ranking strategy to identify the taxa significantly contributing to the differentiation of groups of amplicon metagenomic samples. By integrating the phylogenetic relationships amongst taxa into the framework of statistical learning, the method is able to unambiguously group the taxa into clades without relying on a precompiled taxonomy, and accomplishes a ranking of the clades according to their contribution to the sample differentiation. We applied the method to a meta-analysis of two recent datasets of comparative studies of the gut microbiota of European, USA, African and South American healthy individuals, identifying bacterial taxa that are differentially distributed with geography and age. Comparison of the performances of the method to popular feature selection and classification algorithms shows that or strategy is effective in identifying microbial clades associated to the different sample groups, providing a novel analysis method for targeted metagenomic datasets.

## Results

PhyloRelief is an algorithm that introduces the Relief [21,22] strategy of feature weighting in a phylogenetic context to identify those OTUs or groups of OTUs that are responsible for the differentiation between classes of samples (*i*.*e*. healthy vs. disease, lean vs. obese, population A vs. population B, etc.) in a metagenomic dataset. The method is designed to analyze any set of samples that has been characterized via high throughput sequencing of one or more marker genomic loci, whose sequences have been clustered into OTUs. The process requires that the samples are unambiguously classified into cases and controls according to the description provided by the study design, and that a phylogenetic tree of the OTUs has been obtained by molecular phylogenetic analysis.

The algorithm is composed by two main conceptual steps: i) a scoring scheme that ranks the branches of the OTU tree according to their contribution to the differentiation of the classes, and ii) a merging step that merges nested subtrees into independent clades. At the end of this procedure, PhyloRelief ranks the clades according to their discriminant power between cases and controls.

### Definition of the scores

**Fig 1:**
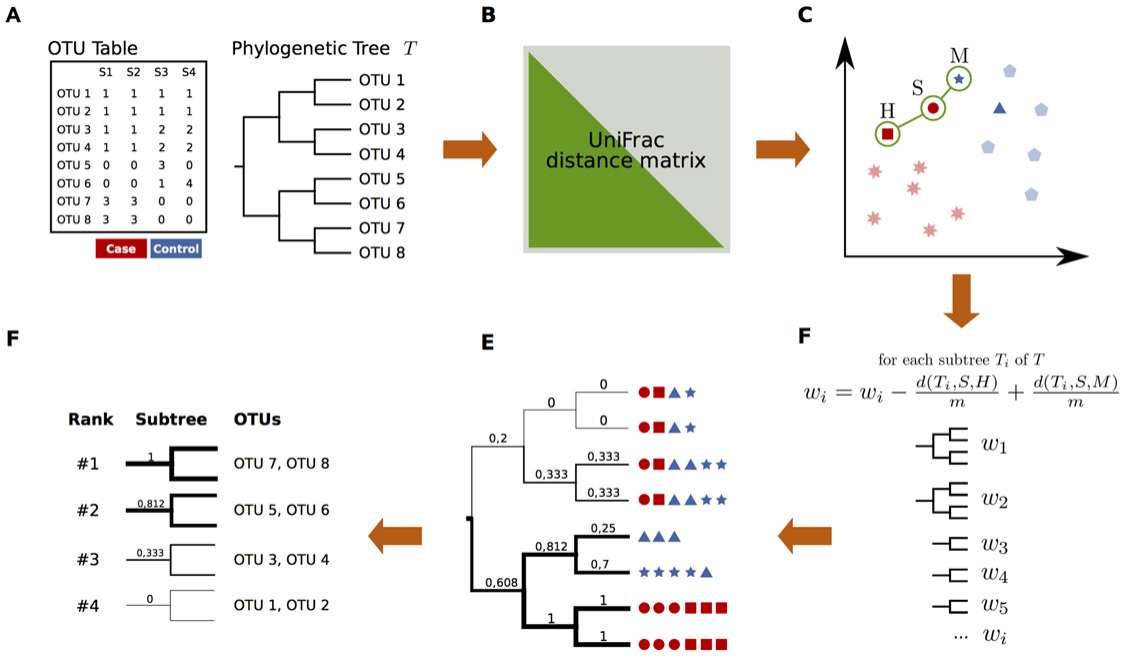
Schema of the method. A) Preliminary analysis. The PhyloRelief algorithm relies on a set of preprocessing steps of the metagenomic datasets that must be performed using standard algorithms. From the sequences of the marker genomic loci selected by the experimental design, an OTU table and a phylogenetic tree of the representative sequences of the OTUs is computed. B) Next, the matrix of the distances between the samples must be computed using a phylogenetic measure of β-diversity, such as weighted or unweighted UniFrac must be provided. C) The PhyloRelief strategy. Once one sample *S* has been randomly selected, the nearest hit *H*, *i*.*e*. the nearest sample of the same class, and the nearest miss *M*, *i*.*e*. the nearest sample of different class according to distance matrix D^S^ are identified. D) The update function. For each subtree T_i_ the weight w_i_ is updated by summing the value *d(T*_*i*_,*S*,*H)/m* and subtracting *d(T*_*i*_,*S*,*M)/m*. The function *d(T*_*i*_,*A*,*B)/m* is computed by summing the UniFrac distance between the sample *A* and *B* restricted to the subtree *T*_*i*_ and *m* is the number of samples. E) Correlation of the weights and definition of the clades. The weights of each clade propagate to the parents, where it is either reinforced if coalescing with a clade sharing similar unbalance between the classes, or is diluted if coalescing with a clade with no or contrasting unbalance. This allows an iterative procedure leading to the unambiguous identification of a set of uncorrelated clades. F) Output. The algorithm provides a list of clades of the phylogenetic tree ranked according to their contribution to the separation of the classes of samples.

Given a partitioning of the samples into two or more classes ({C1}, {C2},…), PhyloRelief ranks the internal branches in the OTU tree by assigning them a score *w* that reflects their importance in the differentiation of the classes. In its simplest form the procedure is as follows. First, one sample *S* is randomly chosen and its nearest hit *H* (*i*.*e*. the nearest sample of the same class) and miss *M* (*i*.*e*. the nearest sample of a different class) are individuated (Fig. 1). Next, the score *w* of each clade is increased by an amount proportional to the contribution of the clade to the distance between *S* and *M*, and decreased by an amount proportional to its contribution to the distance between *S* and *H*. In this way, the score of those clades that support the fact that *S* is more distant from *M* than from *H* is increased, while the score of those that support the contrary is decreased. A detailed description of the update rules is given in the Methods section. After that the procedure has been repeated over all possible choices of *S*, each clade has a score *w* that is high if the clade supports the partitioning of the samples into classes, {C1}, {C2}, and low if it does not (Fig. 1). The critical step of the procedure is the choice of the update function, for which different definitions are possible. Here we define (see Methods): a) an unweighted update function, that, for each clade, is proportional to the fraction of the clade that is unique to one of the classes, *i*.*e*. the fraction of the phylogenetic tree from which descend only OTUs belonging to one of the classes; b) a weighted update function, in which each branch of the tree is weighted by a quantity proportional to its unbalance between the classes, *i*.*e*. the difference between the number of sequences in samples from one class and from the other. Analogously to the Relief-F extension of the Relief algorithm, PhyloRelief can be applied to multi-class problems and can use *k*-nearest neighbors in the score computation, becoming robust in the case of noisy or unbalanced data sets [21].

### Correlation between the lineages and identification of the clades

The peculiar nature of the features that we are ranking (*i*.*e*. subtrees in a tree) introduces a correlation that needs to be taken into account when analyzing the data, and that can be exploited to define a set of independent clades ranking them according to their relevance. If a given branch is heavier, due to unbalanced OTUs distribution between the different classes, its weight will propagate to the parent branches, where it is either reinforced by coalescing with branches sharing a similar unbalance, or diluted if the coalescing branches have contrasting or no unbalances. Exploiting this property, individual lineages can be clustered into taxonomic clades by inspecting the profile of the weights along the tree and identifying the branch where this has a local maximum. This rule, exemplified in S1 Fig, (see Methods) naturally defines a set of independent taxonomic clades and ranks them according to their contribution to the diversification between the classes. Using this ranking, the minimal set of clades necessary to describe the classes to a certain level of accuracy is determined by running non-parametric tests of class diversification, such as PERMANOVA[23] and ANOSIM[24], as a function of the number of clades.

### Applications

**Fig 2:**
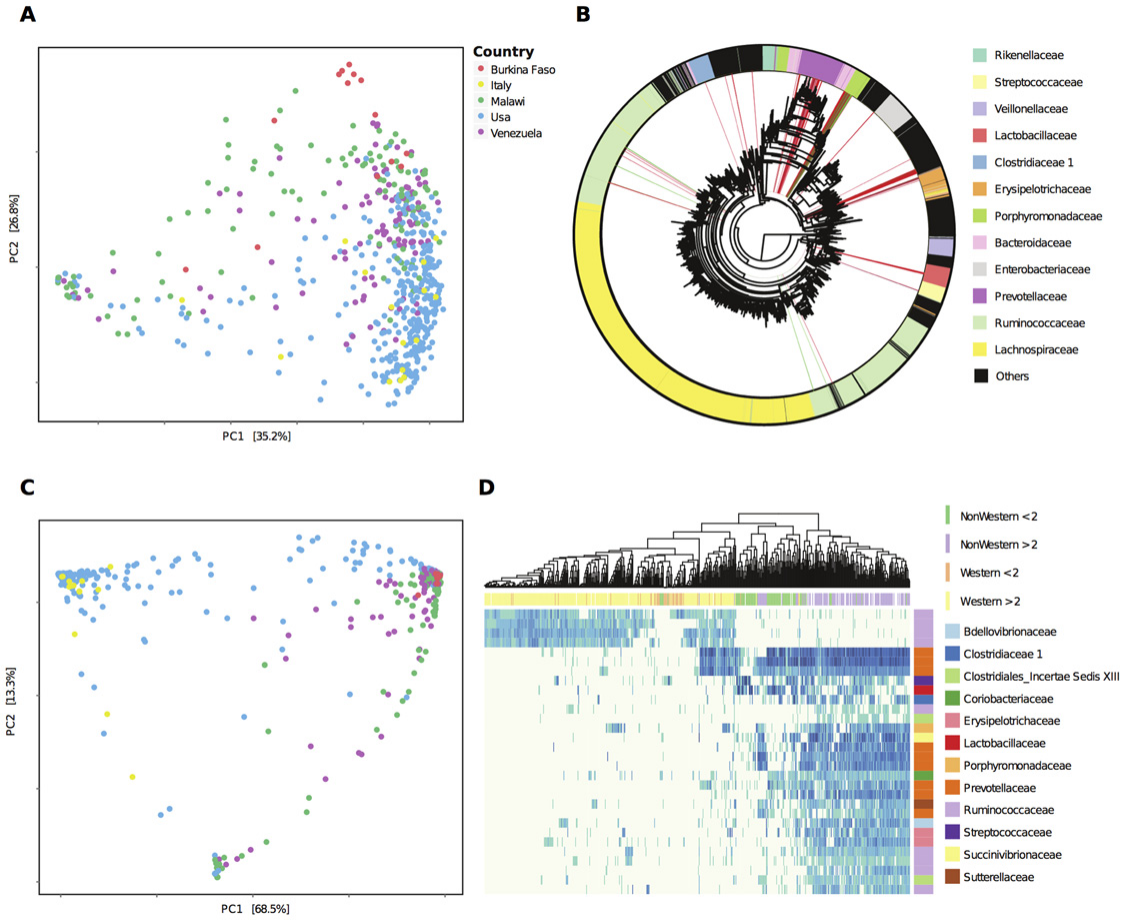
Variability of the gut microbiome with geography. A) PCoA of the weighted UniFrac distances stratified by geographical origin. B) Phylogenetic tree of the OTUs. The 30 clades most relevant for the differentiation of the USA and Italian samples form the Burkina Faso, Malawi and Venezuelan samples are highlighted. Colors distinguish those more prevalent in the USA and Italian samples (green) form those more prevalent in the Burkina Faso, Malawi and Venezuelan samples (red) C) PCoA of the weighted UniFrac distances using only the OTUs included in the most relevant clades. D) Heatmap of the Log_10_ of the relative abundances of the 30 most relevant clades (rows) identified by PhyloRelief. The age and origin of the individuals (columns) are indicated.

In order to illustrate the potentialities of the method, we analyzed two recent datasets, one including 528 samples from healthy individuals of different ages from the United States, from Guhaibo Amerindians living in two villages in Venezuela, and from four rural communities in Malawi [5], and the other including samples from 14 healthy children from the Mossi ethnic group living in a rural setting in Burkina Faso and 15 healthy children living in Florence (Italy)[6]. To allow joint analysis of these two datasets, OTUs were picked using a reference database (see Materials and Methods) and the OTU tables were merged and rarefied to the same number of reads. A PCoA analysis of the weighted UniFrac distances (Fig. 2A) shows that the samples segregate by geographical origin, with the USA and Italian samples clearly distinct from the African (Malawi and Burkina Faso) samples, and the Venezuelan occupying an intermediate position between the two groups. Previous meta-analyses of these data have shown differences in microbiota composition correlating to the “Western” (USA and Italy) or “non-Western” (Malawi, Burkina Faso and Venezuela) origin of the samples, and it has been suggested that these differences are related to the different balance between protein-rich and fiber-rich diet in these communities [2,5,6]. Stratifying the data by age of the subjects shows (S2 Fig) that the age is also an important factor in the variability of the human gut microbiota, and that this variability seems to be highest at younger age.

To identify the taxonomic groups that associate with the geographical origin and that might be correlated to the different diets of the five different populations, we partitioned the samples into two classes, one including the Western subjects (from Italy and the USA), and the other including the non-Western (Malawi, Burkina Faso and Venezuela) subjects, and applied the PhyloRelief algorithm to these two classes. To identify the number of clades that were more relevant to differentiate the two classes, we performed ANOSIM and PERMANOVA analysis with increasing number of clades ranked according to the PhyloRelief weights (Table 1). This procedure showed that both ANOSIM and PERMANOVA had a maximum comprised between 20 and 30 clades, indicating that using this number of clades the separation between the groups is largest. In Fig. 2B we show a phylogenetic tree of the OTUs present in the samples, with those included in the 30 most relevant clades identified by PhyloRelief highlighted (in red OTUs more prevalent in Malawi, Burkina Faso and Venezuela, in green OTUs more prevalent in the USA and Italy). It is worth noting that most of these clades were specifically more represented in the non-Western samples, while only few were specific of the Western individuals, and that much of the differences were confined within the order Bacteroidales. In particular, the Malawi, Burkina Faso and Venezuelan samples were rich in Prevotellaceae, while the Western samples were rich in Ruminococcaceae. In Fig. 2C the PCoA of the weighted UniFrac distances computed on the 30 most relevant clades is shown. Although the Western samples were distinct from the rest, they showed a large degree of variability, with a small fraction of samples from the USA closely related to the Malawi, Burkina Faso and Venezuelan samples. In addition, age was still a major factor, being closely associated to the second component of the PCoA (S3 Fig). To further investigate the individual distribution of the 30 most relevant clades, we show in Fig. 2D a heatmap of the logarithm of the prevalences of the OTUs within these clades. These data confirmed that there was a group of individuals from the USA that were closely related to the non-Western individuals, sharing three clades of Prevotellaceae with most of the Malawi, Burkina Faso and Venezuelan subjects. Stratifying the subjects by age, we found that in both classes young subjects (below 2 years of age) were clearly distinct from older subjects (Fig. 2D, upper panel). In addition, while we found clear separation between Western and non-Western adult subjects, some of the Western young subjects were classified by hierarchical clustering together with the non-Western young subjects and vice-versa, suggesting that at young age cultural or geographical differences are less important in determining the structure of the gut microbiota probably related to the instability of the gut microbiota, a phenomenon typical of childhood[5].

**Table 1:** Permutational ANOVA and ANOSIM tests on the effect of the number of clades used in the calculation of the weighted UniFrac distance between Western (USA and Italy) and non-Western (Malawi, Burkina Faso and Venezuela) individuals.

	PERMANOVA			ANOSIM	
N clades	F	R^2^	p-value	R	p-value
10	49.00	0.13	0.001	0.40	0.001
20	414.44	0.44	0.001	0.68	0.001
30	374.68	0.41	0.001	0.61	0.001
40	139.46	0.20	0.001	0.29	0.001
50	121.25	0.18	0.001	0.27	0.001
60	83.88	0.13	0.001	0.36	0.001
70	82.01	0.13	0.001	0.35	0.001
80	82.24	0.13	0.001	0.35	0.001
90	92.72	0.14	0.001	0.30	0.001
100	86.89	0.14	0.001	0.32	0.001
200	98.98	0.15	0.001	0.36	0.001
300	101.74	0.15	0.001	0.35	0.001
400	109.94	0.17	0.001	0.37	0.001
500	109.73	0.17	0.001	0.37	0.001
600	106.61	0.16	0.001	0.38	0.001
700	94.92	0.15	0.001	0.35	0.001
800	94.90	0.15	0.001	0.35	0.001
900	96.79	0.15	0.001	0.36	0.001
1000	96.51	0.15	0.001	0.36	0.001

**Fig 3:**
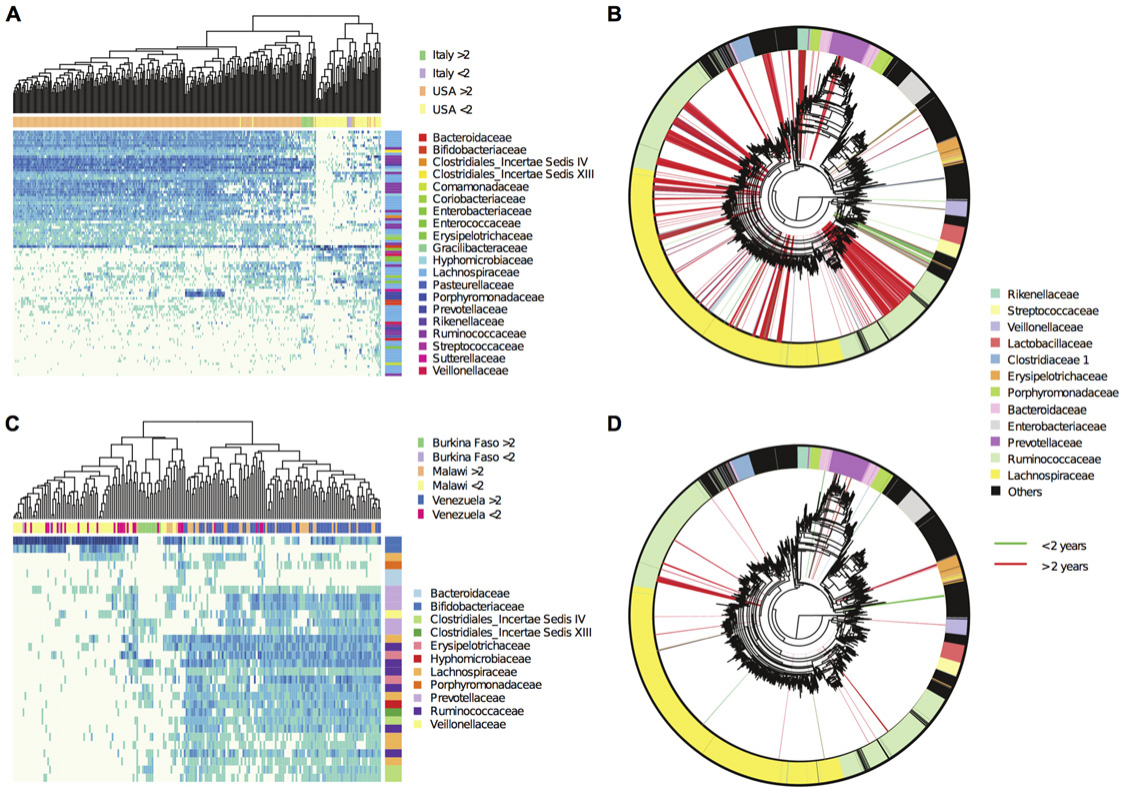
Variability of the gut microbiome with age. A) Italy and USA; Heatmap of the Log_10_ of the relative abundances of the 90 clades (rows) identified by PhyloRelief that differentiate the samples from individuals below 2 years of age from the older individuals (columns). B) Italy and USA; Phylogenetic tree of the OTUs. The 90 most relevant clades are highlighted. Colors distinguish those more prevalent in the younger samples (green) form those more prevalent in the older samples (red). C) Burkina Faso, Malawi and Venezuela; Heatmap of the Log_10_ of the relative abundances of the 30 clades (rows) identified by PhyloRelief that differentiate the samples from individuals below 2 years of age from the older individuals (columns). D) Burkina Faso, Malawi and Venezuela; Phylogenetic tree of the OTUs. The 30 most relevant clades are highlighted. Colors distinguish those more prevalent in the younger samples (green) form those more prevalent in the older samples (red).

To highlight the role of age, and to identify the age for which the differences between young and older individual was highest, we partitioned the samples into two groups using as variable the age threshold, performing a PERMANOVA analysis of the weighted UniFrac distances between the groups as a function of this threshold. We found (S4 Fig and S1 table) that the differentiation between young and older subjects was largest when the age threshold was set to two years, and that above 14 years of age, there was no difference between the microbiome of young and adult subjects. However, running the PhyloRelief analysis on the complete dataset, we could not unambiguously identify a minimal set of bacterial clades associated to this differentiation (S2 Table). This result was likely due to the different gut microbiota of Western and non-Western adult subjects. For this reason, we repeated the analysis separately for Western and non-Western samples. ANOSIM and PERMANOVA showed that the maximum differentiation between individuals below age of 2 and above age of 2 for the Western and for the non-Western samples was achieved using 90 (Table 2) and 30 clades (Table 3), respectively, where both PERMANOVA R^2^ and ANOSIM R have a maximum. The differentiation between young and adults was much sharper in Western subjects, with a prominent role played by Lachnospiraceae and Ruminococcaceae in the adults (Figs. 3A and S5). In non-Western subjects (Figs. 3B and S6), there was also a contribution of the presence of five clades of Prevotellaceae to the differentiation of the adult gut microbiota. In both Western and non-Western samples the younger subjects have higher abundance of Bifidobacteriaceae (Fig. 3), probably due to breast-feeding in infants [5]. Bifidobacteriaceae were present at lower prevalence in most adult subjects, except for the adults from Burkina Faso probably due to the absence of dairy food in adult age in this African population [25].

**Table 2:** Permutational ANOVA and ANOSIM tests on the effect of the number of clades used in the calculation of the weighted UniFrac distance between young (below two years of age) and older (above two years of age) Western individuals (USA and Italy).

	PERMANOVA			ANOSIM	
N clades	F	R^2^	p-value	R	p-value
**10**	138.89	0.31	0.001	0.75	0.001
**20**	50.38	0.14	0.001	0.58	0.001
**30**	92.73	0.22	0.001	0.73	0.001
**40**	76.12	0.19	0.001	0.71	0.001
**50**	72.53	0.18	0.001	0.69	0.001
**60**	69.68	0.18	0.001	0.68	0.001
**70**	72.43	0.18	0.001	0.71	0.001
**80**	78.38	0.20	0.001	0.74	0.001
**90**	124.58	0.28	0.001	0.81	0.001
**100**	121.48	0.27	0.001	0.80	0.001
**110**	117.01	0.27	0.001	0.80	0.001
**120**	110.17	0.26	0.001	0.78	0.001
**130**	110.45	0.26	0.001	0.78	0.001
**140**	110.62	0.26	0.001	0.78	0.001
**150**	111.04	0.26	0.001	0.78	0.001
**160**	111.04	0.26	0.001	0.78	0.001
**170**	107.45	0.25	0.001	0.78	0.001
**180**	107.89	0.25	0.001	0.77	0.001
**190**	103.66	0.24	0.001	0.76	0.001
**200**	101.49	0.24	0.001	0.75	0.001
**200**	101.49	0.24	0.001	0.75	0.001
**300**	88.90	0.22	0.001	0.74	0.001
**400**	85.75	0.21	0.001	0.72	0.001
**500**	88.47	0.22	0.001	0.72	0.001
**600**	88.89	0.22	0.001	0.73	0.001
**700**	89.97	0.22	0.001	0.73	0.001
**800**	89.82	0.22	0.001	0.73	0.001
**900**	89.79	0.22	0.001	0.73	0.001
**1000**	89.77	0.22	0.001	0.73	0.001

**Table 3:** Permutational ANOVA and ANOSIM tests on the effect of the number of clades used in the calculation of the weighted UniFrac distance between young (below two years of age) and older (above two years of age) non-Western individuals (Malawi, Burkina Faso and Venezuela).

	PERMANOVA			ANOSIM	
N clades	F	R^2^	p-value	R	p-value
**10**	17.54	0.09	0.001	0.38	0.001
**20**	18.92	0.10	0.001	0.42	0.001
**30**	173.33	0.47	0.001	0.73	0.001
**40**	154.84	0.45	0.001	0.72	0.001
**50**	161.55	0.46	0.001	0.72	0.001
**60**	144.83	0.43	0.001	0.68	0.001
**70**	125.26	0.39	0.001	0.66	0.001
**80**	129.25	0.40	0.001	0.66	0.001
**90**	111.63	0.37	0.001	0.63	0.001
**100**	111.37	0.37	0.001	0.60	0.001
**110**	111.52	0.37	0.001	0.60	0.001
**120**	103.80	0.35	0.001	0.59	0.001
**130**	103.00	0.35	0.001	0.58	0.001
**140**	103.52	0.35	0.001	0.59	0.001
**150**	94.73	0.33	0.001	0.53	0.001
**160**	93.17	0.33	0.001	0.53	0.001
**170**	92.03	0.32	0.001	0.53	0.001
**180**	91.97	0.32	0.001	0.53	0.001
**190**	91.39	0.32	0.001	0.53	0.001
**200**	91.20	0.32	0.001	0.54	0.001
**200**	91.20	0.32	0.001	0.54	0.001
**300**	85.45	0.31	0.001	0.52	0.001
**400**	78.85	0.29	0.001	0.51	0.001
**500**	73.88	0.28	0.001	0.46	0.001
**600**	69.12	0.26	0.001	0.46	0.001
**700**	69.20	0.26	0.001	0.46	0.001
**800**	69.12	0.26	0.001	0.46	0.001
**900**	68.97	0.26	0.001	0.46	0.001
**1000**	68.92	0.26	0.001	0.46	0.001

### Predictivity of the ranked features in supervised classification problems

The main goal of supervised classification is to build a model from a set of labeled samples to classify new, uncategorized data in high dimensional datasets in the presence of complex relationships between the variables. Identifying a ranking strategy to reduce the dimensionality of the dataset can improve the effectiveness of classification algorithms in metagenomic datasets, where correlations between the variables are introduced both by the phylogenetic relationships between the clades and by the fact that relative abundances are measured. The Random Forest (RF) classifier was recently proven to be the most effective in this class of problems [26,27], both for feature selection and classification. Although the main goal of this work is to define a phylogeny-based OTUs ranking method, it is interesting to assess the predictive power of the ranked taxa for the classification of samples into predefined categories in comparison to other state of the art algorithms. For this purpose, we selected four publicly available datasets, including data from four body sites (forehead vs. external nose and volar forearm vs. popliteal fossa) [1], from fecal samples (IBD vs. healthy) [28] and skin [29] (using both subject identification—3 classes—and subject/hand identification—6 classes—as target) that have recently been used as benchmark in comparative evaluations of classification methods for metagenomic data [26,27,28].

We compared the performance of PhyloRelief coupled with the RF classifier to LEfSe [30], an algorithm that uses statistical tests for biomarker discovery, to MetaPhyl, a recent phylogeny-based method for the classification of microbial communities [31] and to Random Forest, used both as classifier and feature selection method. The performances were assessed in terms of average predictive accuracy using the K-category correlation coefficient (KCCC), a multiclass extension of the Matthews Correlation Coefficient (MCC) [32] (see Materials and Methods for details on the procedure). The results are reported in Table 4. We found that while in one case (in the volar forearm vs. popliteal fossa sample) the OTUs identified by LEfSe had a higher predictive value, in all other cases PhyloRelief coupled to RF performed equivalently to the most efficient alternative algorithm (FH vs. EN: Phylorelief 0.220+/-0.073—MetaPhyl 0.170+/-0.106; IBD: Phylorelief 0.213+/-0.074—LEfSe 0.238+/-0.065; FS subject: PhyloRelief 1.0—LEfSe 1.0; FS subject/hand: PhyloRelief 0.684+/-0.026—RF 0.670+/-0.026), suggesting that taxa identified using phylogenetic information have high predictive power in classification problems.

**Table 4:** Classification accuracy in terms of average K-category correlation coefficient (KCCC) using weighted and unweighted PhyloRelief, LEfSe using OTUs and classified taxa, RF and MetaPhyl.

		FH vs. EN (CBH)	VF vs. PF (CBH)	IBD	FS subject (C = 3)	FS subject/hand (C = 6)
**PhyloRelief W + RF**	k = 2	0.214 0.103 (4)	0.655 0.045 (800)	-0.011 0.060 (40)	**1 0 (700)**	0.678 0.028 (900)
	k = 3	0.158 0.060 (4)	0.718 0.033 (800)	0.079 0.090 (40)	**1 0 (700)**	0.666 0.027 (800)
	k = 4	**0.220 0.073 (4)**	0.685 0.065 (800)	0.074 0.067 (40)	**1 0 (700)**	**0.684 0.026 (900)**
**PhyloRelief U + RF**	k = 2	-0.042 0.087 (4)	0.565 0.077 (800)	0.165 0.057 (40)	**1 0 (700)**	0.655 0.024 (900)
	k = 3	0.112 0.095 (4)	0.539 0.080 (800)	0.213 0.074 (40)	0.994 0.006 (700)	0.640 0.020 (800)
	k = 4	0.066 0.089 (4)	0.599 0.050 (800)	0.121 0.078 (40)	0.994 0.006 (700)	0.653 0.017 (900)
**LEfSe + RF**	OTU	-0.039 0.061 (19)	**0.836 0.040 (100)**	0.083 0.057 (81)	**1 0 (181)**	0.628 0.022 (59)
	Taxa	0.044 0.059 (4)	0.833 0.035 (50)	**0.238 0.065 (20)**	0.983 0.008 (85)	0.517 0.034 (101)
**RF**	FS	0.108 0.099 (1)	0.784 0.074 (40)	0.142 0.059 (7)	1.0 0.0 (200)	0.670 0.026 (30)
	No FS	-0.021 0.021 (-)	0.659 0.060 (-)	0.0 0.0 (-)	1.0 0.0 (-)	0.667 0.026 (-)
**MetaPhyl**	No FS	0.170 0.106 (-)	0.831 0.048 (-)	0.229 0.085 (-)	0.950 0.022 (-)	0.672 0.036 (-)

For PhyloRelief, three value of k (k = 2,3,4) are shown. When feature selection was performed using PhyloRelief, LEfSe and RF, the RF classifier was used. For each of these algorithms we report the cross-validation accuracy in terms of average KCCC, the Standard Error and the number of features selected in the final model using the complete dataset (in parentheses). For PhyloRelief and RF the number of features was selected by a nested cross validation loop. For each dataset, the maximum KCCC value is marked in bold.

## Discussion

High throughput sequencing applied to the study of microbial communities is revolutionizing the way we understand the role of microorganisms in the environment and in health and disease conditions. The relatively low cost of sequencing has triggered an exponential increase in the amount of data generated, that have highlighted correlations between the structure of the microbiota and important human pathologies for which conventional intervention strategies were not effective. This suggests that a precise definition of the structure of the healthy vs. disease microbiota could allow early diagnosis and the definition of effective intervention strategies in a number of pathologies. To become a viable diagnostic and therapeutic tool, the evolution of sequencing technologies needs to be paralleled by progress in computational tools enabling to significantly correlate phenotypes to the smallest possible number of microbial taxa. This would allow, on one hand, to develop relatively cheap and easy to use diagnostic tools, and on the other hand to design focused and personalized intervention strategies.

PhyloRelief is an algorithm that resolves the problem of relevant taxa identification by applying the Relief strategy of feature ranking in a phylogenetic context. The improvement of this method over existing ones consists in its ability to accomplish a ranking of the microbial clades, defined on the basis of the taxa distribution amongst the samples weighted by phylogenetic information, discovering those that contribute to the differentiation between two or more classes of samples. Importantly, this result is obtained without relying on a predefined set of taxonomic categories that are often hard pressed to describe the complexity of the evolutionary relationships between microorganisms.

We applied the algorithm to case control studies derived from the literature, in all cases identifying taxa that are significantly differentially distributed. Of particular interest were the results obtained when comparing infants vs. adults in the different geographies, showing that age has a much greater influence in the USA and Italy than in the African and South American samples, with a much larger fraction of the OTU differentially distributed between young children and adults in the former than in the latter. Comparing the performances of the algorithm to LEfSe, MetaPhyl and to Random Forest in a classical supervised classification schema using cross validation, we found that the taxa ranked by PhyloRelief had also a high predictive value, performing as well as—and in some cases outperforming—current gold standard methods.

The algorithm is general and does not rely on any specific sequencing technology, as long as a phylogenetic tree of the OTUs and the distribution of the OTUs in the different samples are available. The method presented here is technology agnostic since it can be used to interpret data generated by the targeted amplification of marker genomic loci, such as the variable regions of the 16S rDNA gene for bacteria, or the ITS sequences for fungi as well as complete metagenome sequencing data, such as those obtained with Illumina technologies. In addition, the algorithm can readily be extended to regression problems to include cases where a continuous variable differentiate the individual samples using the RReliefF extension of Relief [21,22]. The PhyloRelief class of algorithms fills a significant gap in the growing array of computational methods that are currently used for the analysis of metagenomic data, and will impact importantly on the application of metagenomics to the development of novel diagnostic markers, leading the application of these approaches from the bench to the bedside.

## Materials and Methods

### The PhyloRelief algorithm

We will assume that a phylogenetic tree *T* of the OTUs is given, and that a distance matrix *D* ^*S*^ between the samples S has been computed according to some measure of β -diversity. Given the availability of phylogenetic data, β -diversity measures incorporating phylogenetic information, such as weighted and unweighted UniFrac [19,20] have become popular in the context of metagenomic research, but other measures, such as Bray-Curtis dissimilarity index could also be used. Let us define a partitioning of *S* into sample class {*C1*} and {*C2*}. Usually, this partitioning is obtained either by exploratory analysis of the distance matrix *D* ^*S*^, or by the study design (*e*.*g*. according to the origin of the samples, health status or age of the donor in the case of human samples, etc.).

The purpose of the PhyloRelief algorithm is to rank the OTUs according to their relevance in the partitioning of *S* into {*C1*} and {*C2*}. To accomplish this result, we developed a modified version of the Relief-F procedure that takes into account the phylogenetic information contained in the tree *T*. To this purpose, the algorithm does not work directly with the OTUs, but with the clades (or sub-trees) *T*_*i*_ of the tree *T*. Below we report the two main steps of the algorithm, i.e. i) the scoring scheme ranking the sub-trees of the tree *T*, and ii) the merging step that identifies the independent clades.

#### Definition of the scores

Two different scoring function have been define: a unweighted update function and a weighted update function. Being closely related to the unweighted UniFrac distance, the unweighted update function is the natural choice when exploratory analysis of the samples has been performed using unweighted UniFrac, while the weighted update function is the natural choice when weighted UniFrac has been used.

Unweighted PhyloRelief. For each subtree *T*_*i*_ (1≤*i*≤*N*)of *T*, we compute a weight w[*T*_*i*_] with the following iterative procedure:

**Start**

 define the subtrees *T*_*i*_ (1≤*i*≤*N*) that have the branches *B*_*i*_ as root;

 set all weights w[*T*_*i*_] = 0;

**for** j: = 1 **to** *m* **do** begin

  randomly select a sample *S*_*j*_;

  find nearest hit *H* and nearest miss *M*;

**for** *i* **=** 1**to** *N* **do**

$$w\left[T_{i}\right]=w\left[T_{i}\right]-\frac{d\left(T_{i},S_{j},H\right)}{m}+\frac{d\left(T_{i},S_{j},M\right)}{m}$$

**end;**

**end;**

The function *d*(*T*_*i*_,*A*,*B)* is equal to:

$$d\left(T_{i},A,B\right)=\frac{\sum_{B_{q}\in \left\{T_{i}\right\}}b_{q}\left|{\text{Θ}}_{q}^{A}-{\text{Θ}}_{q}^{B}\right|}{\sum_{B_{q}\in \left\{T_{i}\right\}}b_{q}}$$

where $\Theta_{q}^{S}$ is equal to 1 if the branch *B*_*q*_ contains OTUs from sample *S*, 0 otherwise. In this way, the contribution of each clade does not take into account the prevalence of the OTUs in the different classes, but just their presence. Consequently, the algorithm identifies those lineages that are specific to one of the classes of samples.

**Weighted PhyloRelief**. In weighted PhyloRelief we use the same iterative procedure defined above, but the update function *d*(*T*_*i*_,*A*,*B)* is defined as:

$$d\left(T_{i},A,B\right)=\frac{\sum_{B_{q}\in \left\{T_{i}\right\}}b_{q}\left|p_{q}^{A}-p_{q}^{B}\right|}{\sum_{B_{q}\in \left\{T_{i}\right\}}b_{q}\left(p_{q}^{A}+p_{q}^{B}\right)}$$

The sum runs on all branches *B*_*q*_ of *T*_*i*_ (including *B*_*i*_). $p_{q}^{A}$ and $p_{q}^{B}$ are the fraction of the taxa descending from the branch *B*_*q*_ that are from samples *A* and *B*, respectively. *b*_*q*_ is the length of the branch *B*_*q*_. In other words, *d*(*T*_*i*_,*A*,*B)* is the weighted UniFrac distance between samples *A* and *B* due to the OTUs in the subtree *T*_*i*_ of the tree *T* defined by the branch *B*_*i*_. By iterating over *S*_*j*_, the procedure positively weights those sub-trees *T*_*i*_ that support the partitioning of *S* into {*C1*} and {*C2*}, and negatively those that do not support the partitioning. *m* is a user defined parameter that, in practice, can be set to the number of samples in *S*.

#### Generalizations and extensions

Analogously to the Relief-F algorithm, PhyloRelief can work with multi-class classification problems. Moreover, in its generalized form, the algorithm again randomly selects a sample *S*, but then identifies *k* of its nearest neighbors from the same class, called nearest hits *H*_*l*_, and *k* nearest neighbors from each of the different classes, called nearest misses *M*_*l*_(*C*).

**Start**

 define the subtrees *T*_*i*_ (1≤*i*≤*N*) that have the branches *B*_*i*_ as root;

 set all weights w[*T*_*i*_] = 0;

**for** j: = 1 **to** *m* **do begin**

  randomly select a sample *S*_*j*_;

  find *k* nearest hits *H*_*l*_;

**for** each class *C≠class(S*_*j*_) **do**

   find *k* nearest misses *M*_*l*_(*C*);

**for** *i* = 1**to** *N* **do**

$$w\left[T_{i}\right]=w\left[T_{i}\right]-\sum_{l=1}^{k}\frac{d\left(T_{i},S_{j},H_{l}\right)}{m*k}+\sum_{C\neq class\left(S_{j}\right)}\frac{P\left(C\right)}{1-P\left(class\left(S_{j}\right)\right)}\;\sum_{l=1}^{k}\frac{d\left(T_{i},S_{j},M_{l}\right)}{m*k}$$

**end;**

**end;**

**end;**

where *P*(*C*) is the fraction of samples in class *C*. The factor $\frac{P\left(C\right)}{1-P\left(class\left(S_{j}\right)\right)}$ is required for ensuring appropriate normalization and to guarantee that the contribution of hits and of each class of misses is between 0 and 1.

#### Definition of the clades

Let *T*_*j*_ be a sub-tree of *T*_*i*_. The correlation between the values *w*[*T*_*i*_], *w*[*T*_*j*_] is illustrated by the example in S1 Fig, where the weights for four samples (“circles”, “squares”, “triangles” and “stars”) partitioned into two classes (“red” and “blue”) are shown. The high weights in the bottom clade (Clade b, containing OTUs only from the “red” class of samples) propagates up from the terminal branches until it merges with Clade a, that contains OTUs from the “blue” class of samples. In the parent branch of Clades **a** and **b**, the unbalance between the two samples is diluted, and consequently the weight decreases. In this example, Clade **a** and clade **b** separately, but not their parent clade, would be responsible for the differentiation between “red” and “blue” samples.

In order to exploit the information contained in the weights *w*[*T*_*i*_], it is crucial to define a set of independent clades and rank those in order of importance. To accomplish this, we identify the sub-tree *T* with the highest weight. In the case of ties, we randomly start from one of the subtrees if these are independent, or take the one closest to the root if these are nested. This defines the first clade. Next, we prune the tree from all the branches descending from *T*, and from those ascending from *T*. This last step is needed to avoid the possibility to iteratively enlarge the same clades, given the correlation between the weights of nested sub-trees discussed above. Iteratively applying this rule, we define a set of independent clades ranked according to their weight. Using this ranking, the minimal set of clades necessary to describe the classes to a certain level of accuracy is determined by running non-parametric tests of class diversification, such as PERMANOVA[23] and ANOSIM[24], as a function of the number of clades. Alternatively, univariate non-parametric tests such as the Kruskal—Wallis could be applied for testing whether samples originate from the same distribution in each clade.

#### Applications

Definition of the OTU table. USA, Venezuela and Malawi dataset. Reads were downloaded from the MG-RAST web server (http://metagenomics.anl.gov/metagenomics.cgi?page=MetagenomeProject&project=401). II) Burkina vs. European children. Raw data were obtained from the Sequence Read Archive (http://www.ncbi.nlm.nih.gov/sra), study number ERP000133. Forward and reverse primers were removed using cutadapt [33] and reads missing the forward primer were discarded. Quality trimming was performed by sickle (https://github.com/najoshi/sickle), with quality threshold 20. Reads with length <100 were discarded. Chimeras were removed by uchime[34] (reference database mode) using the Greengenes[35] database (Release 13_5) clustered at 85% identity threshold as reference.

OTUs were picked using QIIME (pick_otus.py seqs.fna --max_accepts 1 --max_rejects 8 --stepwords 8 -- word_length 8 -C) against the Greengenes database clusters at 97% identity level (Greengenes database available at: http://greengenes.lbl.gov/Download/Sequence_Data/Fasta_data_files/Reference_OTUs_for_Pipelines/Caporaso_Reference_OTUs/gg_otus_4feb2011.tgz). The OTU table was obtained using custom scripts, and rarefied using the rarefy_even_depth() function in the phyloseq package (version 1.6.1) of the R (version 3.0.2) statistical software. Taxonomy was assigned to the representative sequences using the RDP classifier [36] (version 2.5) with a confidence threshold of 0.8. UniFrac distances were computed and the PhyloRelief analysis was performed using the reference phylogenetic tree from the Greengenes release.

IBD, Costello et al. Body Habitats (CBH) and Fierer et al. Subject (FS) Datasets. Preprocessed reads and metadata were downloaded from the Qiime repository http://www.microbio.me/qiime/ under the study ids 1290, 449 and 232 respectively. OTU tables and phylogenetic trees were inferred using the standard QIIME pipeline with default settings, where OTUs were picked with UCLUST [37] at a sequence similarity threshold of 0.97%. Taxonomy was assigned using the Greengenes database (version 2013/05). Rarefactions were performed at the depth of the shallowest sample. Phylogenetic tree were computed using PyNAST [38] (using the Greengenes 2013/05 database as template) and FastTree [39].

#### Non parametric tests

Permutational ANOVA (PERMANOVA) and ANOSIM tests were performed with 999 permutations.

### Predictive classification pipeline

We compared the predictive performance of PhyloRelief with the Random Forest classifier (PhyloRelief +RF) to LEfSe + RF, MetaPhyl (without feature selection) and Random Forest used as both classifier and feature selection method (RF + RF).

To assess the prediction performance of the weighted features we implemented a predictive pipeline based on a stratified 10x random subsampling cross validation (CV). Data are partitioned into a training set and a testing set (75% and 25% of the samples respectively). In order to avoid overfitting and selection bias effects, the feature selection procedure was included in the cross validation loop [40,41]. For each training set, the number of ranked features *n*_0_ that provides the smallest average KCCC is found by a nested 10x random subsampling CV. Later, the features are ranked using the entire training set and the model is trained using the top ranked *n*_0_ features. The model is finally tested on the independent testing set and a KCCC is computed. In the case of LEfSe + RF, LEfSe was treated as feature selection method using the common p-value threshold of 0.05. For MetaPhyl, no feature selection was performed and the nested CV was used to find the optimal model parameters (parameters grid: *λ* = {100000, 1000, 100, 10, 1, 0.1, 0.01, 0.001, 0.0001} and *w* = {0, 0.2, 0.4, 0.6, 0.8, 1}). For the Random Forest classifier, the number of trees was set to 500 and the weights were computed as in [42]. In PhyloRelief OTUs were ranked using the weights computed on the related clades.

The pipeline was developed in Python using the scikit-learn module (http://scikit-learn.org).

#### Software dependencies and availability

PhyloRelief is implemented in Python (http://www.python.org), and requires Python > = 2.7 with the NumPy/SciPy (http://www.scipy.org), Pandas (http://pandas.pydata.org/), DendroPy [43] and Statsmodels (http://statsmodels.sourceforge.net/) libraries. PhyloRelief software, scripts and data analyzed in this paper are available at http://compmetagen.github.io/phylorelief.

## Supporting Information

S1 Fig The PhiloRelief scoring scheme In this example, four samples (“circles”, “squares”, “triangles” and “stars”) are partitioned into two classes (“red” and “blue”). The Bottom clades (Clade **a** and Clade **b**) have high weight since they contain OTUs only from the “blue” and “red” class of samples, respectively. The higher weights of the branches in clade **b** take into account the more even distribution of the “blue” class of samples. The weights propagate up from the terminal branches until the two clades merge. In the parent branch of Clade **a** and Clade **b** the unbalance between the two samples is diluted, and consequently the weight decreases.
(TIF)

S2 Fig PCoA of the weighted UniFrac distances stratified by age. While the first component is correlated to geography (see Fig. 2) the second is related to the age of the subjects.
(TIF)

S3 Fig PCoA of the weighted UniFrac distances computed using only the OTUs included in the 30 most relevant clades for the differentiation between Western (Italy and USA) and non-Western (Malawi, Burkina Faso and Venezuela) populations. Colors indicate the age of the subjects. Above two years (red); below two years (blue).
(TIF)

S4 Fig R^2^ of the Permutational ANOVA obtained by partitioning the individuals into two samples using an increasing age threshold, and the pair-wise weighted UniFrac distance. The differentiation is maximum for two years of age, and there is no difference between the two sub-samples above 16 years.
(TIF)

S5 Fig PCoA of the weighted UniFrac distances between Western (Italy and USA) individuals using only the OTUs included in the 90 most relevant clades. Colors indicate the age of the subjects. Above two years (red); below two years (blue).
(TIF)

S6 Fig PCoA of the weighted UniFrac distances between non-Western (Malawi, Burkina Faso and Venezuela) individuals using only the OTUs included in the 30 most relevant clades. Colors indicate the age of the subjects. Above two years (red); below two years (blue).
(TIF)

S1 Table Permutational ANOVA and ANOSIM tests on the effect of age. The sample has been partitioned into two as function of an age threshold and the Permutational ANOVA and ANOSIM tests have been computed using the weighted UniFrac distance.
(DOCX)

S2 Table Permutational ANOVA and ANOSIM tests on the effect of the number of clades used in the calculation of the UniFrac distance between young (below two years of age) and older (above two years of age) individuals. (DOCX)
