# Supplementary figures and images for: Explaining Diversity in Metagenomic Datasets by Phylogenetic-Based Feature Weighting
Source: PLoS Comput Biol. 2015 Mar 27;11(3):e1004186. doi: 10.1371/journal.pcbi.1004186 (PMC4376673; doi:10.1371/journal.pcbi.1004186)

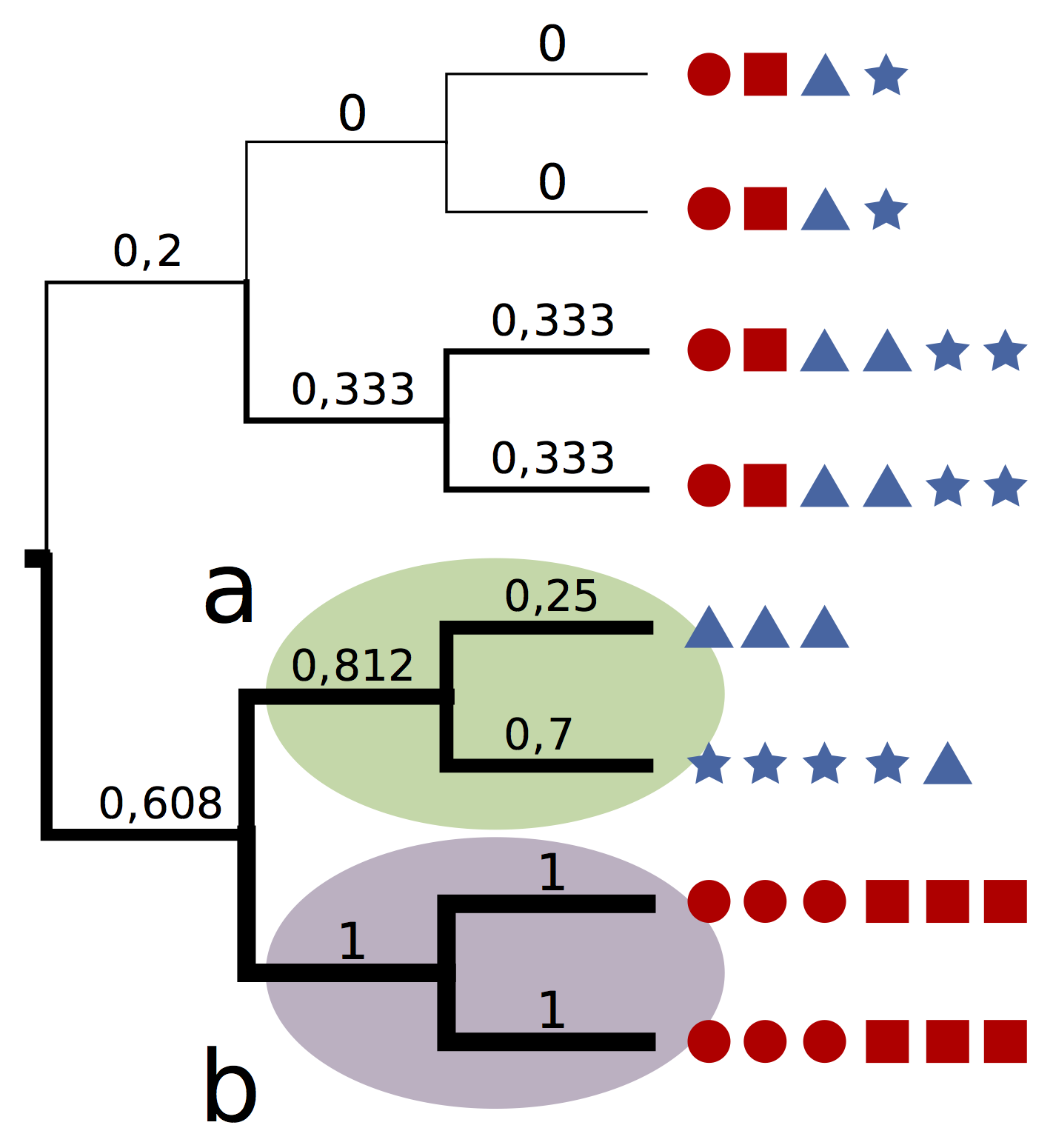

Supplement: S1 Fig — In this example, four samples (“circles”, “squares”, “triangles” and “stars”) are partitioned into two classes (“red” and “blue”). The Bottom clades (Clade a and Clade b) have high weight since they contain OTUs only from the “blue” and “red” class of samples, respectively. The higher weights of the branches in clade b take into account the more even distribution of the “blue” class of samples. The weights propagate up from the terminal branches until the two clades merge. In the parent branch of Clade a and Clade b the unbalance between the two samples is diluted, and consequently the weight decreases. (TIF) [file pcbi.1004186.s001.tif]

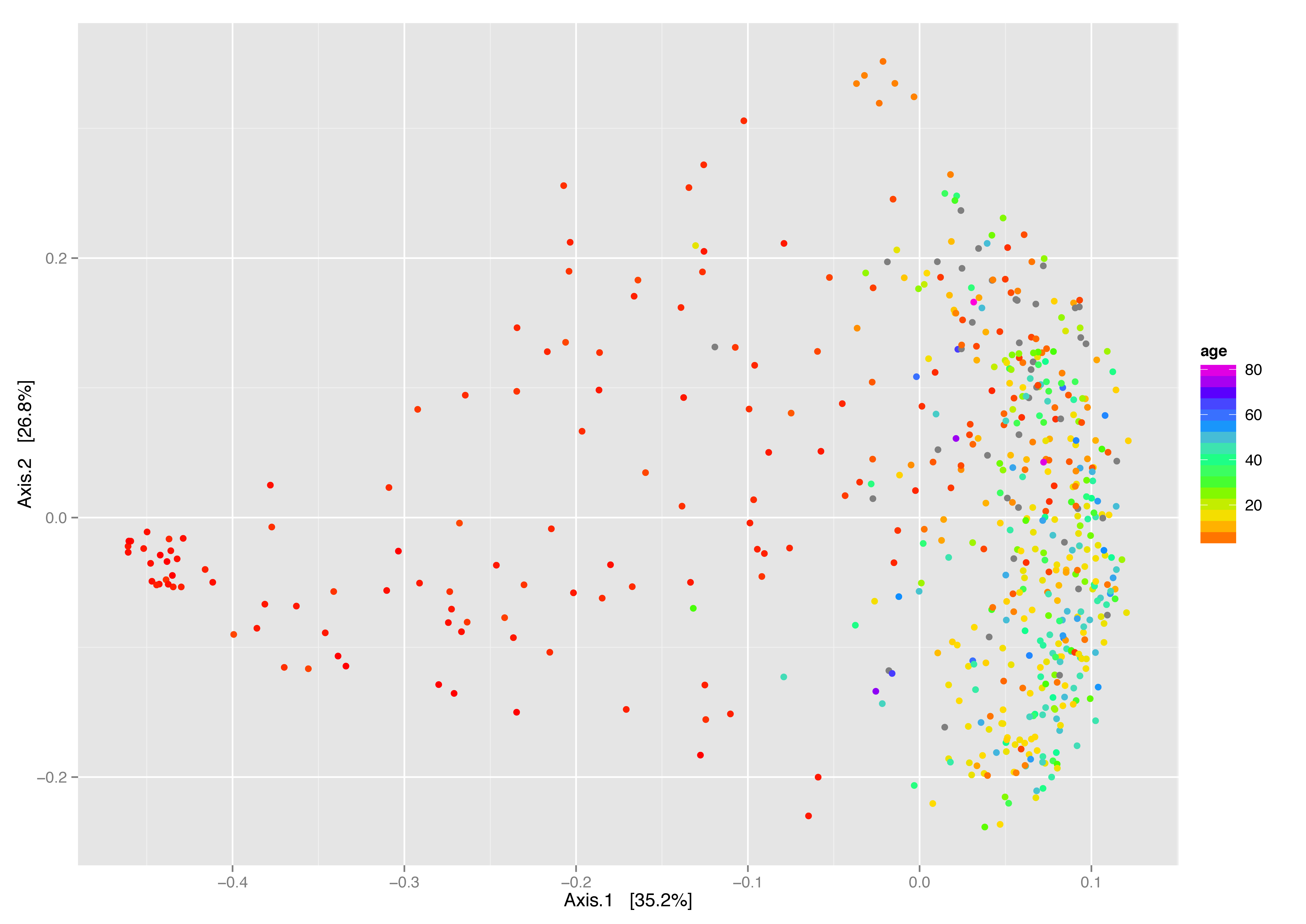

Supplement: S2 Fig — While the first component is correlated to geography (see Fig. 2) the second is related to the age of the subjects. (TIF) [file pcbi.1004186.s002.tif]

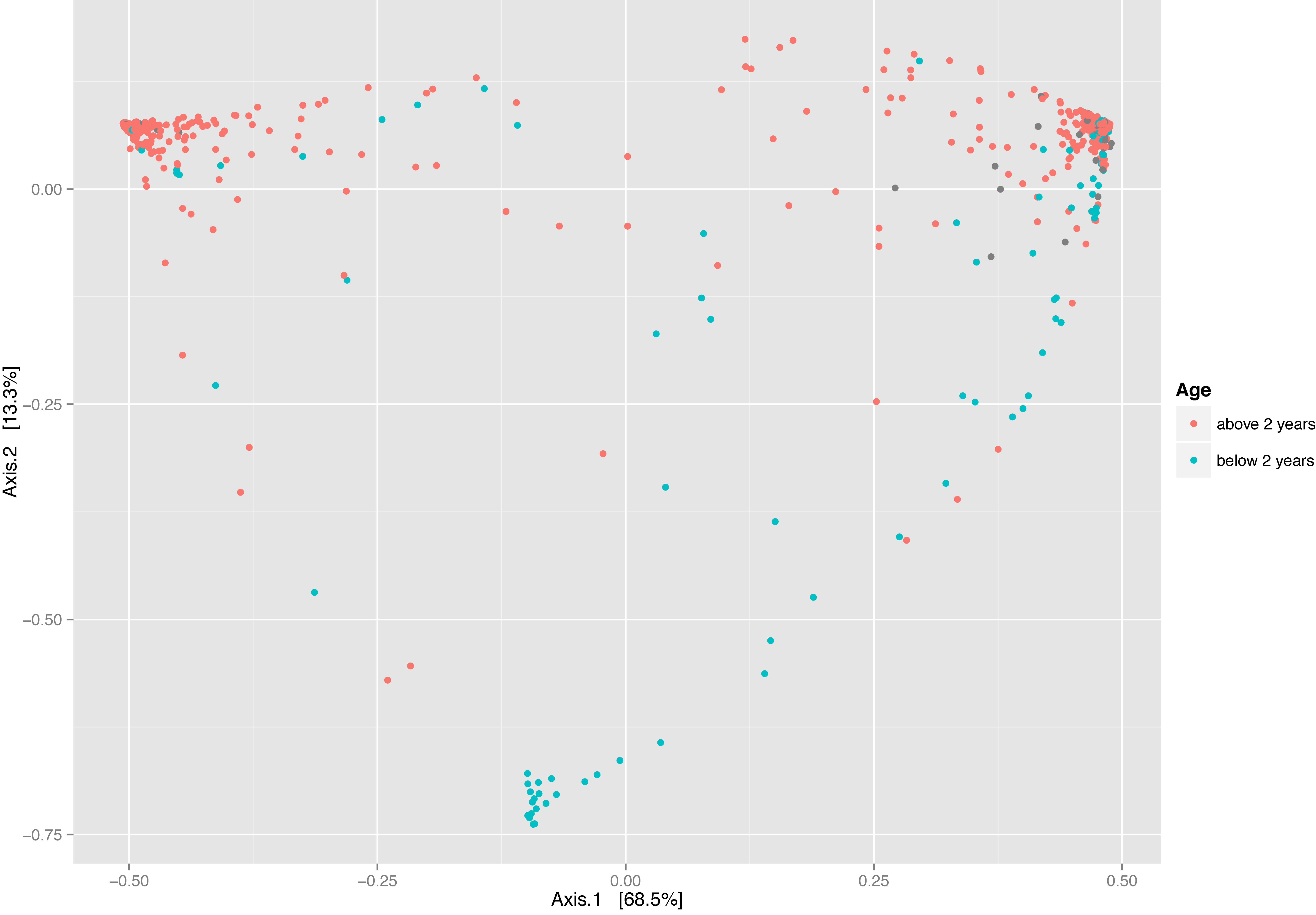

Supplement: S3 Fig — Colors indicate the age of the subjects. Above two years (red); below two years (blue). (TIF) [file pcbi.1004186.s003.tif]

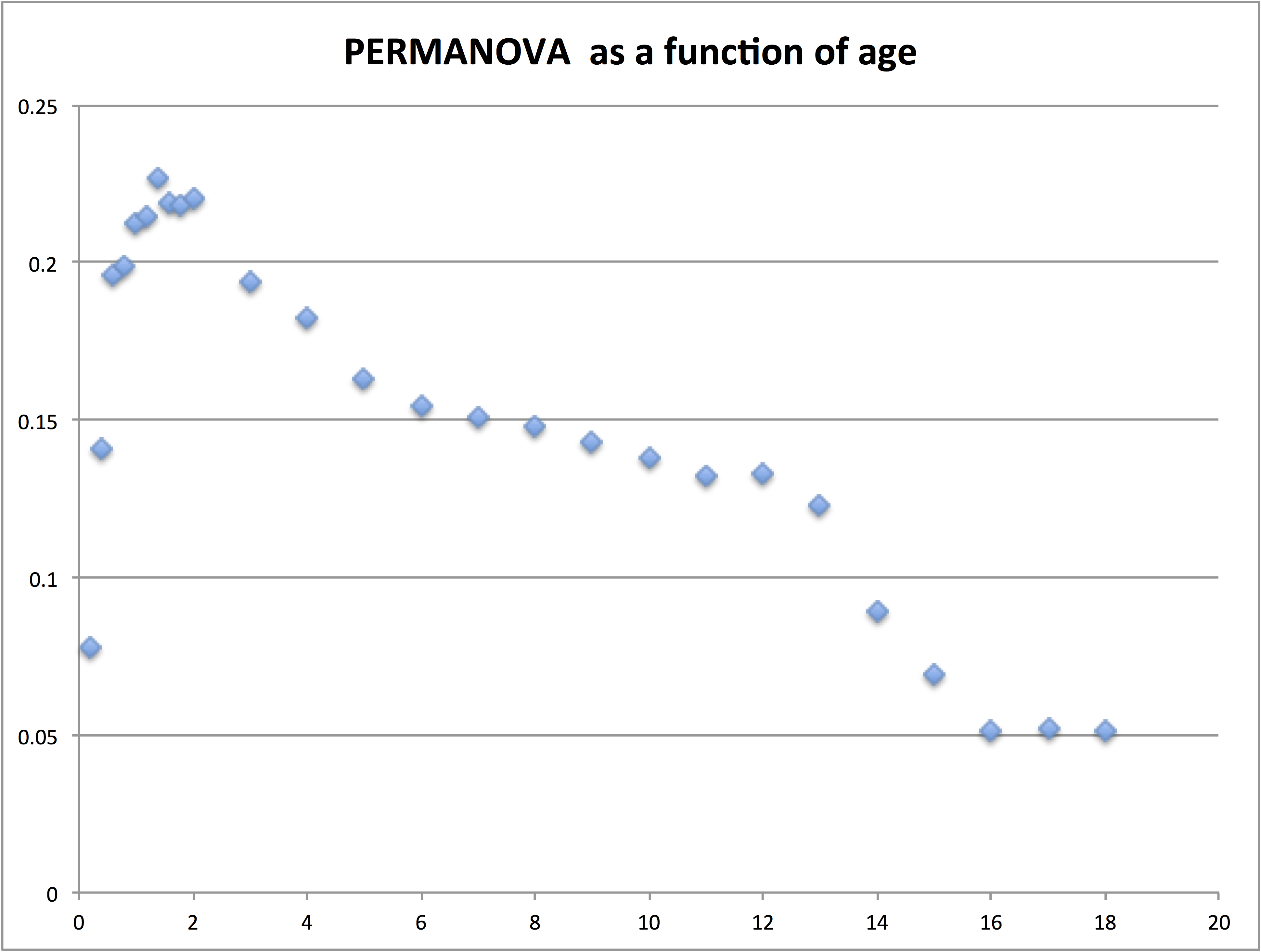

Supplement: S4 Fig — The differentiation is maximum for two years of age, and there is no difference between the two sub-samples above 16 years. (TIF) [file pcbi.1004186.s004.tif]

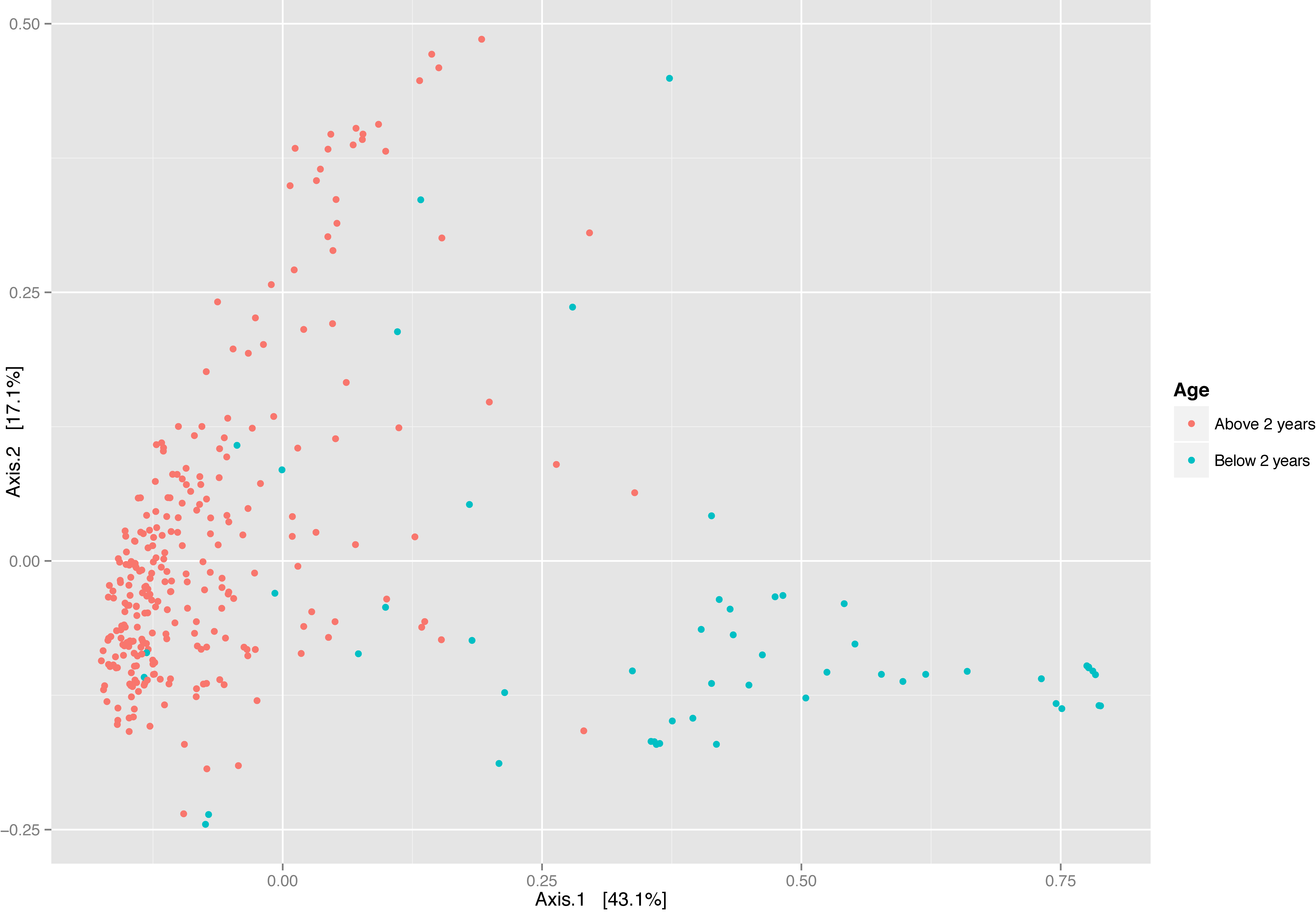

Supplement: S5 Fig — Colors indicate the age of the subjects. Above two years (red); below two years (blue). (TIF) [file pcbi.1004186.s005.tif]

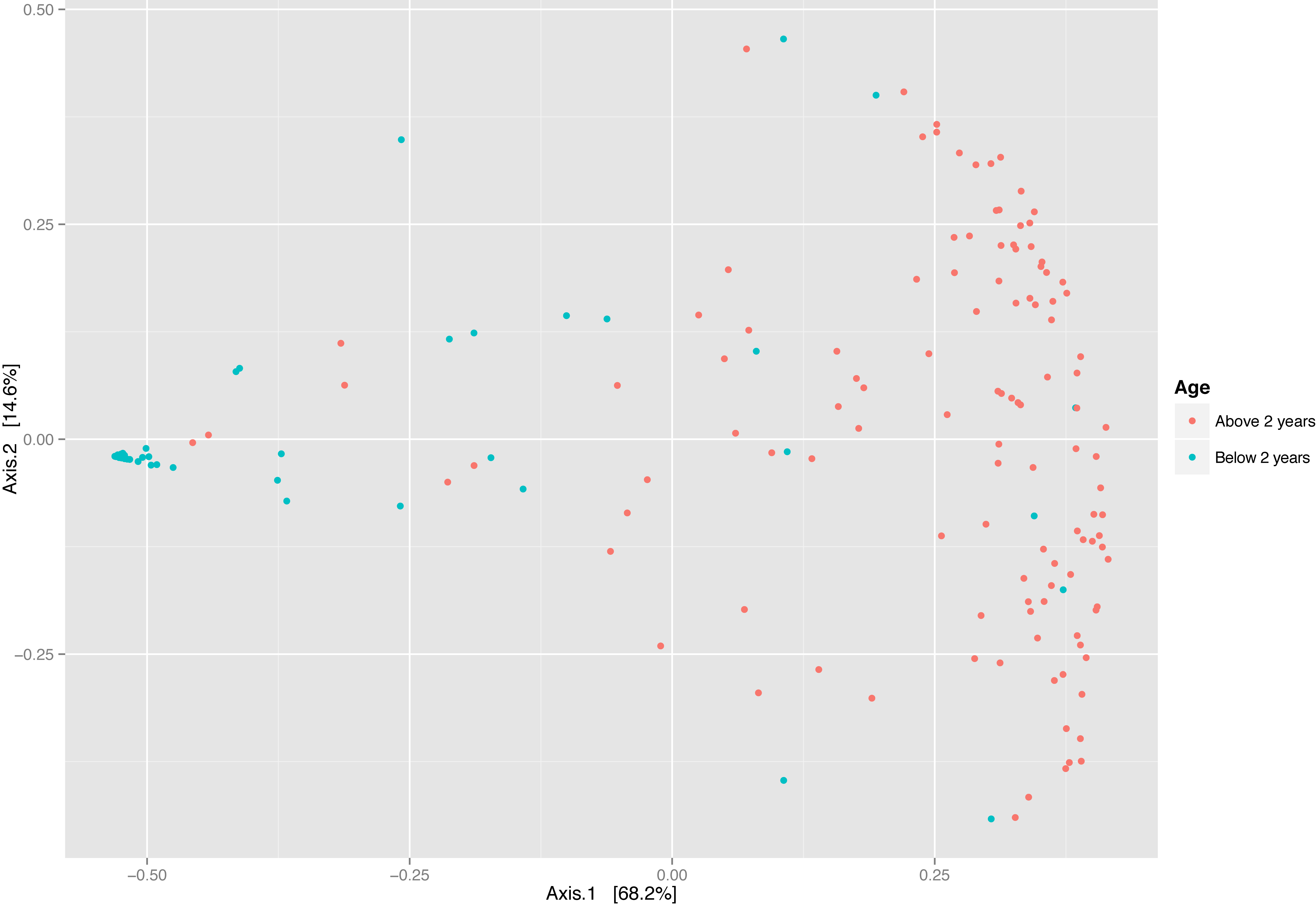

Supplement: S6 Fig — Colors indicate the age of the subjects. Above two years (red); below two years (blue). (TIF) [file pcbi.1004186.s006.tif]
