# Supplementary material for: Explaining Diversity in Metagenomic Datasets by Phylogenetic-Based Feature Weighting
Source: PLoS Comput Biol. 2015 Mar 27;11(3):e1004186. doi: 10.1371/journal.pcbi.1004186 (PMC4376673; doi:10.1371/journal.pcbi.1004186)
Supplement: S1 Table — The sample has been partitioned into two as function of an age threshold and the Permutational ANOVA and ANOSIM tests have been computed using the weighted UniFrac distance. (DOCX) [file pcbi.1004186.s007.docx]

|  | PERMANOVA | | | Anosim | |
| --- | --- | --- | --- | --- | --- |
| Age (years) | F | R^2^ | p-val | R | p-val |
| 0.2 | 43.60 | 0.08 | 0.001 | 0.76 | 0.001 |
| 0.4 | 84.63 | 0.14 | 0.001 | 0.78 | 0.001 |
| 0.6 | 125.39 | 0.20 | 0.001 | 0.75 | 0.001 |
| 0.8 | 127.63 | 0.20 | 0.001 | 0.70 | 0.001 |
| 1 | 138.71 | 0.21 | 0.001 | 0.65 | 0.001 |
| 1.2 | 140.99 | 0.21 | 0.001 | 0.65 | 0.001 |
| 1.4 | 151.18 | 0.23 | 0.001 | 0.66 | 0.001 |
| 1.6 | 144.15 | 0.22 | 0.001 | 0.64 | 0.001 |
| 1.8 | 143.70 | 0.22 | 0.001 | 0.63 | 0.001 |
| 2 | 145.80 | 0.22 | 0.001 | 0.61 | 0.001 |
| 3 | 123.92 | 0.19 | 0.001 | 0.52 | 0.001 |
| 4 | 115.09 | 0.18 | 0.001 | 0.49 | 0.001 |
| 5 | 100.13 | 0.16 | 0.001 | 0.43 | 0.001 |
| 6 | 94.24 | 0.15 | 0.001 | 0.40 | 0.001 |
| 7 | 91.68 | 0.15 | 0.001 | 0.38 | 0.001 |
| 8 | 89.58 | 0.15 | 0.001 | 0.37 | 0.001 |
| 9 | 85.72 | 0.14 | 0.001 | 0.34 | 0.001 |
| 10 | 82.47 | 0.14 | 0.001 | 0.32 | 0.001 |
| 11 | 78.36 | 0.13 | 0.001 | 0.29 | 0.001 |
| 12 | 79.21 | 0.13 | 0.001 | 0.29 | 0.001 |
| 13 | 72.22 | 0.12 | 0.001 | 0.24 | 0.001 |
| 14 | 50.36 | 0.09 | 0.001 | 0.10 | 0.001 |
| 15 | 38.44 | 0.07 | 0.001 | 0.02 | 0.02 |
| 16 | 27.91 | 0.05 | 0.001 | -0.04 | 0.998 |
| 17 | 28.22 | 0.05 | 0.001 | -0.05 | 1 |
| 18 | 28.04 | 0.05 | 0.001 | -0.05 | 1 |
