# Supplementary material for: Explaining Diversity in Metagenomic Datasets by Phylogenetic-Based Feature Weighting
Source: PLoS Comput Biol. 2015 Mar 27;11(3):e1004186. doi: 10.1371/journal.pcbi.1004186 (PMC4376673; doi:10.1371/journal.pcbi.1004186)
Supplement: S2 Table — (DOCX) [file pcbi.1004186.s008.docx]

|  | PERMANOVA | | | Anosim | |
| --- | --- | --- | --- | --- | --- |
| N of clades | F | R^2^ | p-val | R | p-val |
| 10 | 210.21 | 0.29 | 0.001 | 0.61 | 0.001 |
| 20 | 124.41 | 0.20 | 0.001 | 0.61 | 0.001 |
| 30 | 107.04 | 0.17 | 0.001 | 0.55 | 0.001 |
| 40 | 141.79 | 0.22 | 0.001 | 0.63 | 0.001 |
| 50 | 138.53 | 0.21 | 0.001 | 0.63 | 0.001 |
| 60 | 131.11 | 0.20 | 0.001 | 0.62 | 0.001 |
| 70 | 129.54 | 0.20 | 0.001 | 0.62 | 0.001 |
| 80 | 140.58 | 0.21 | 0.001 | 0.62 | 0.001 |
| 90 | 119.67 | 0.19 | 0.001 | 0.61 | 0.001 |
| 100 | 115.32 | 0.18 | 0.001 | 0.59 | 0.001 |
| 200 | 161.82 | 0.24 | 0.001 | 0.64 | 0.001 |
| 300 | 147.99 | 0.22 | 0.001 | 0.61 | 0.001 |
| 400 | 150.47 | 0.23 | 0.001 | 0.61 | 0.001 |
| 500 | 151.33 | 0.23 | 0.001 | 0.62 | 0.001 |
| 600 | 149.61 | 0.23 | 0.001 | 0.62 | 0.001 |
| 700 | 142.74 | 0.22 | 0.001 | 0.61 | 0.001 |
| 800 | 143.37 | 0.22 | 0.001 | 0.61 | 0.001 |
| 900 | 144.90 | 0.22 | 0.001 | 0.61 | 0.001 |
| 1000 | 144.90 | 0.22 | 0.001 | 0.61 | 0.001 |
